# Construction Notes

**Published:** 1905-04-29

**Authors:** 


### CONSTRUCTION NOTES.

It is proposed to erect a new Workhouse Infirmary at Steyning.

The erection of a Municipal Sanatorium for the treatment of Consumptives is under discussion at Belfast.

A sum of £6,000 has already been promised to establish a consumption hospital for Dumfries; £2,000 is still wanting.

The Urban Council of Conway and Penmaenmawr and the Conway Rural Council have unanimously accepted a site at Brynhyfryd, offered by the Corporation, for a joint isolation hospital.

